# The Marine-Derived Sipholenol A-4-*O*-3′,4′-Dichlorobenzoate Inhibits Breast Cancer Growth and Motility *in Vitro* and *in Vivo* through the Suppression of Brk and FAK Signaling

**DOI:** 10.3390/md12042282

**Published:** 2014-04-14

**Authors:** Mohamed R. Akl, Ahmed I. Foudah, Hassan Y. Ebrahim, Sharon A. Meyer, Khalid A. El Sayed

**Affiliations:** 1 Department of Basic Pharmaceutical Sciences, School of Pharmacy, University of Louisiana, 1800 Bienville Drive, Monroe, LA 71201, USA; E-Mails: mohamedreda_pharmacy@yahoo.com (M.R.K.); A_foudah@hotmail.com (A.I.F.); hassanyahia_1982@yahoo.com (H.Y.E.); 2 Department of Toxicology, School of Pharmacy, University of Louisiana, 1800 Bienville Drive, Monroe, LA 71201, USA; E-Mail: meyer@ulm.edu

**Keywords:** breast cancer, Brk, FAK, migration, invasion, sipholane triterpene

## Abstract

Sipholenol A is a natural sipholane triterpenoid isolated from the Red Sea sponge, *Callyspongia siphonella*. Previous studies showed the antimigratory and antiproliferative activities of the semisynthetic sipholenol A esters against breast cancer cell lines. This study investigated the effects of sipholenol A-4-*O*-3′,4′-dichlorobenzoate (SPA) on the growth, migration and invasion of diverse human breast cancer cells. Results showed that SPA inhibited the growth of the human breast cancer cells, MDA-MB-231, MCF-7, BT-474 and T-47D, in a dose-dependent manner. Immunofluorescent analysis showed that SPA significantly reduced Ki-67-positive cells in MDA-MB-231 cells. Flow cytometry and Western blot analyses revealed that SPA treatment suppressed MDA-MB-231 cell growth by inducing cell cycle arrest at the G1 phase. In addition, SPA suppressed breast cancer cell migration, invasion and decreased Brk and FAK activation in a dose-dependent manner. Molecular docking study suggested a perfect fitting at the FAK’s FERM domain, inhibiting the main autophosphorylation site, Y397, which was further confirmed by Western blot analysis. Most known small molecule FAK inhibitors target the kinase domain, creating several off-target side effects. The *in vivo* studies showed that SPA treatment suppressed breast tumor growth and Ki-67, CD31, p-Brk and p-FAK expression in orthotopic breast cancer in nude mice. In conclusion, SPA inhibited the growth, invasion and migration of breast cancer cells possibly via deactivating Brk and FAK signaling, suggesting good potential for therapeutic use to control invasive breast cancer.

## 1. Introduction

Despite advances in medical and surgical therapy, breast cancer annually kills more than 40,000 women in the United States, and the majority of these patients succumb to metastatic disease [1,2]. Tumor cell migration is part of the metastatic cascade, in which tumor cells invade, migrate and leave the primary tumor to gain access to the circulation and reach distal secondary sites [3]. Breast tumor kinase (Brk), an intracellular non-receptor tyrosine kinase, which belongs to the Src family tyrosine kinases, is overexpressed in up to 86% of invasive human breast tumors [4,5,6]. Brk has a significant role in the promotion of cancer cell proliferation, migration and invasion. Brk promotes signaling through EGFR, HER2, insulin-like growth factor-1R, MET and integrins. Brk shares several direct substrates with Src, including paxillin and focal adhesion kinase (FAK), indicating possible redundant functions [4,5,6]. Importantly, Brk and FAK phosphorylation (activation) can consequently lead to AKT, paxillin and MAPK activation [7,8]. FAK is a highly phosphorylated protein localized in focal adhesion contacts of cells [8,9]. Its activity is regulated by integrin-mediated cell adhesion, as well as the activation of growth factor receptor and G-protein-linked receptor signaling [8,9]. FAK is involved in various cell functions, including proliferation, survival, motility, invasion and metastasis [8]. FAK tyrosine Y397 is the main autophosphorylation site of FAK leading to the activation of its intrinsic kinase function, as well as downstream signaling molecules [8,9]. The interaction of Y397-activated FAK and Src causes tyrosine phosphorylation of multiple sites in FAK (-576, -577, -925), as well as signaling molecules, such as paxillin, which connects FAK to various intracellular signaling molecules, such as Ras/MAPK, PI_3_K/AKT and Rho family GTpases [7,8]. Both Brk and FAK have been associated with increased invasion and migration of tumors; hence, inhibiting these redundant pathways has been proposed as a strategy to block the invasive growth of cancer cells [7].

**Figure 1:**
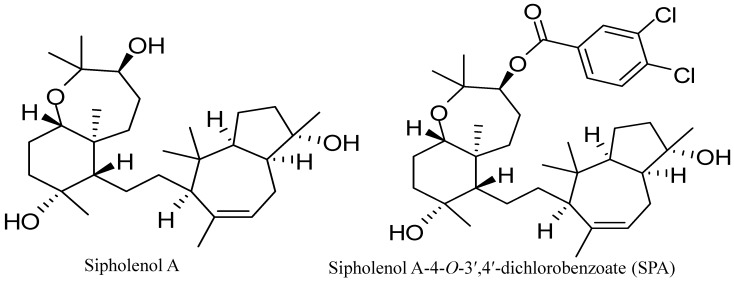
Chemical structures of sipholenol A and sipholenol A-4-*O*-3′,4′-dichlorobenzoate (SPA).

The traditional approach to treat invasive breast cancer has been the use of cytotoxic chemotherapy, which in most cases associated with toxicity and resistance [2]. Therefore, recent efforts focused on developing entities that have selective and specific molecular target(s) in cancer cells in order to improve efficacy and limit toxicity [2]. Marine-derived natural products are among the unique resources for anticancer drug discovery [10]. Sipholenol A (Figure 1), a sipholane triterpenoid, was isolated from the Red Sea sponge, *Callyspongia* (*Siphonochalina*) *siphonella* [11]. Semisynthetic analogs of sipholenol A have been prepared to improve its antimigratory and antiproliferative activities against different breast cancer cell lines [12,13]. Recently, sipholenol A-4-*O*-3′,4′-dichlorobenzoate (SPA) (Figure 1) showed the most potent anticancer activity with no toxicity on the non-tumorigenic human epithelial breast cells [13]. In addition, it resulted in a marked inhibition of the phosphorylation of Brk in a cell-free Z′-LYTE™ kinase assay (Life Technologies, Grand Island, NY, USA) and Western blot analysis in the human breast cancer cells MDA-MB-231 [13].

The aim of this study is to analyze the effects of SPA on key human breast cancer cell lines. The cell lines in this study were chosen as they are the representative of a wide range of breast cancer phenotypes. ERα, one of the most important targets in human breast cancer therapy, is expressed in MCF-7, BT-474 and T-47D cells, whereas MDA-MB-231 cells lack expression of ERα. EGFR is expressed in T-47D, BT-474 and MDA-MB-231 cells. HER-2 is expressed at lower levels in the ER-negative cell line, MDA-MB-231, and expressed at higher levels in the ER-positive cell lines, MCF-7, BT-474 and T-47D. Met is expressed in MDA-MB-231, MCF-7 and BT-474, while it is absent in T-47D cells. Therefore, the four human breast cancer cell lines (MBA-MD-231, MCF-7, BT-474 and T-47D) were chosen to identify the mechanism of action by which SPA inhibits breast cancer cell proliferation and motility and the potential involvement of Brk and FAK signaling. In addition, a xenograft orthotopic breast cancer nude mouse model was used to evaluate the *in vivo* activity of SPA*.*

## 2. Results

### 2.1. Effect of SPA on Human Breast Cancer Cell Growth

**Figure 2:**
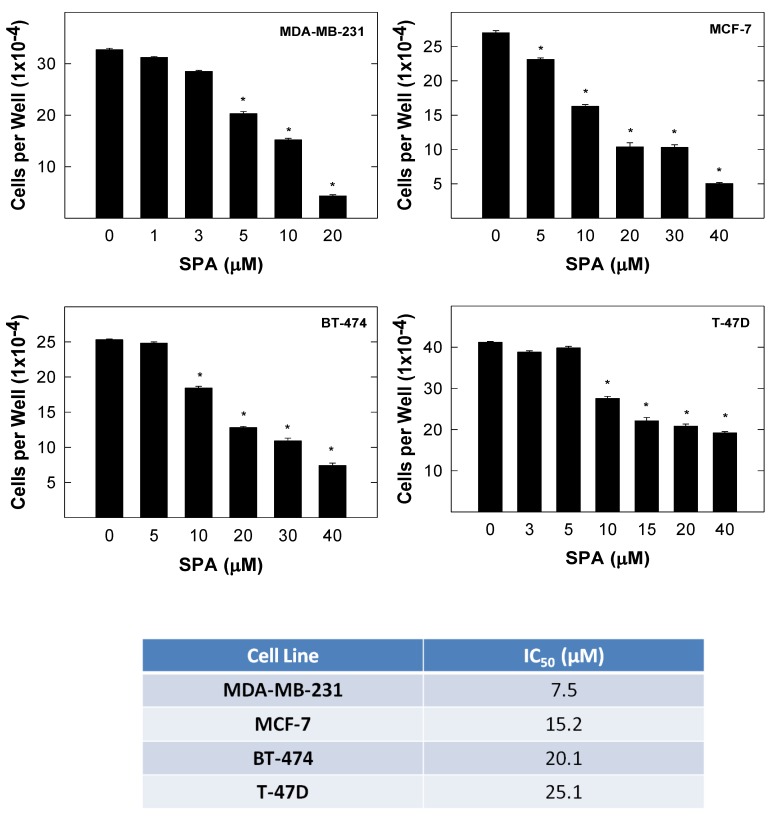
The effect of SPA on the growth of MDA-MB-231, MCF-7, BT-474 and T-47D human breast cancer cells after a 72-h treatment period. The viable cell count was determined by the 3-(4,5-dimethylthiazol-2yl)-2,5-diphenyl tetrazolium bromide (MTT) assay. Vertical bars indicate the mean cell count ± SEM in each treatment group. * *p* < 0.05 as compared with vehicle-treated controls.

The antiproliferative effects of SPA on the growth of various breast cancer cells after a 72-h culture period are shown in Figure 2. Treatment with SPA inhibited MDA-MB-231, MCF-7, BT-474 and T-47D cell growth in a dose-dependent manner as compared to cells in the vehicle-treated control groups (Figure 2). The IC_50_ values for SPA were 7.5, 15.2, 20.1 and 25.1 μM in MDA-MB-231, MCF-7, BT-474 and T-47D breast cancer cells, respectively. 

### 2.2. Effect of SPA on Ki-67 Labeling and Cell Cycle Progression in Human MDA-MB-231 Breast Cancer Cells

**Figure 3:**
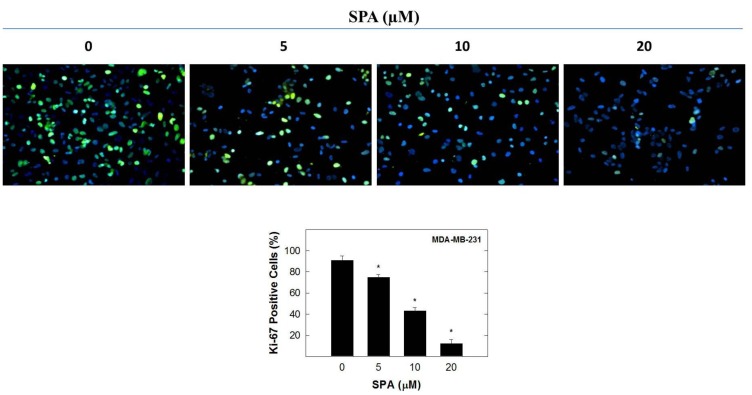
The effect of SPA on Ki-67 staining in MDA-MB-231 cancer cells after a 72-h treatment period. The percentage of actively dividing MDA-MB-231 cancer cells within each treatment group is determined by counting cells with positive Ki-67 staining (green) as a proportion of the total number of cells (DAPI staining, blue). The images here show merged Ki-67 and DAPI. Vertical bars represent the mean ± SEM in each treatment group. * *p* < 0.05 as compared with vehicle-treated control group.

Positive Ki-67 staining is a marker for proliferating cells [14,15]. Positive Ki-67 labeling was observed in 91% of MDA-MB-231 cancer cells grown in control media containing 20 ng/mL of EGF after a 72-h culture period (Figure 3). Treatment with five, 10, and 20 μM of SPA resulted in a 17.5, 52.7 and 86.8% reduction in Ki-67 nuclear staining compared to the vehicle-treated control group (Figure 3).

**Figure 4:**
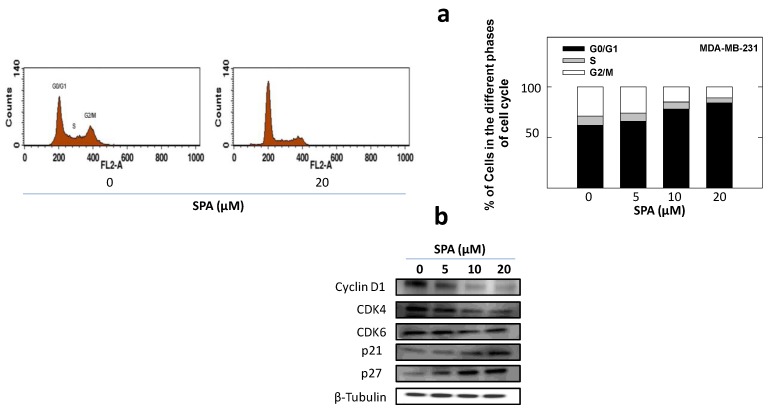
The effect of SPA treatment on EGF-induced G_1_/S cell cycle progression in human MDA-MB-231 breast cancer cells. (**a**) Flow cytometry analysis for cell cycle progression in control and SPA-treated MDA-MB-231 cells. The left panel shows histograms generated using CellQuest software (PI staining) (BD Biosciences, San Jose, CA, USA). The right panel shows the percentage of cells in each phase of the cell cycle. (**b**) Western blot analysis showing the SPA treatment effect on G_1_/S cell cycle regulator proteins.

The effects of SPA treatment on cell cycle progression was evaluated using flow cytometry and Western blot analysis (Figure 4). MDA-MB-231 cells exposed to various doses of SPA resulted in a dose-dependent increase in the proportion of cells in the G_0_/G_1_ phase of the cell cycle from 62% (vehicle-treated control) to nearly 84% with the 20 μM of SPA treatment (Figure 4a). Additional studies were conducted to determine the effects of SPA treatment on the relative intracellular levels of cyclins, cyclin-dependent kinases (CDKs) and cyclin-dependent kinase inhibitors (CKIs), as determined by Western blot analysis (Figure 4b). Treatment with SPA resulted in a prominent reduction in cyclin D1 and CDK4 levels as compared to the vehicle-treated control group (Figure 4b). However, the treatment of MDA-MB-231 cells with 5–20 μM of SPA had little effect on the relative levels of CDK6 (Figure 4b). In addition, SPA treatment caused a marked increase in CKI proteins p21 and p27, compared to the vehicle-treated controls (Figure 4b).

### 2.3. Effects of SPA Treatment on Human Breast Cancer Cell Migration and Invasion

**Figure 5:**
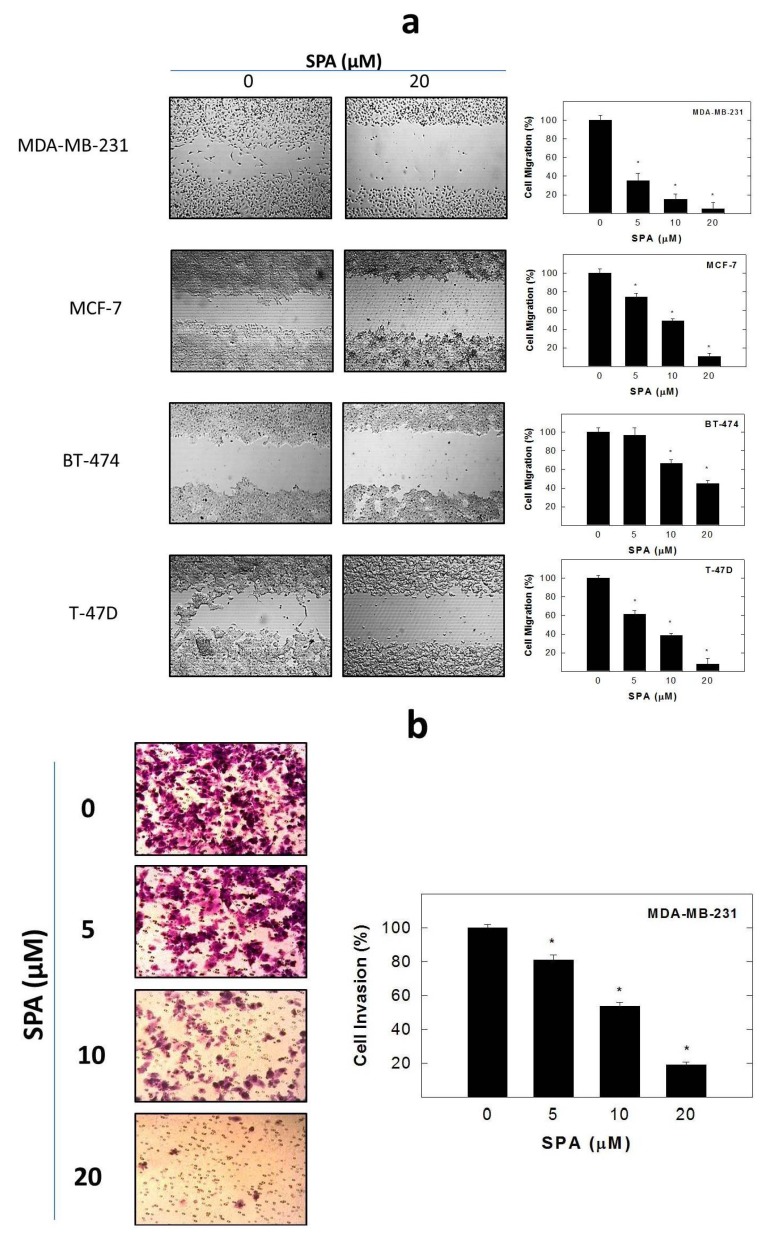
The effect of SPA on breast cancer cell migration and invasion. (**a**) Wound healing assay. The left panel represents photomicrographs of the wound healing assay showing that SPA treatment inhibited the migration of MDA-MB-231, MCF-7, BT-474 and T-47D cancer cells. The right panel shows the quantitative analysis of the percentage of cell migration in various treatment groups. Vertical bars indicate thepercentage of cell migration at 48 h after wounding was calculated relative to the wound distance at time 0 (*t*_0_) ± SEM in each treatment group. * *p* < 0.05 as compared with vehicle-treated control. (**b**) Transwell invasion chamber assay. The cells were treated with 5, 10, and 20 μM SPA for 24 h. Cells invading the basement membrane were analyzed at the end of the treatment period. The left panel represents photomicrographs of the cell invasion assay showing that SPA treatment suppressed the invasion of MDA-MB-231 cells. The right panel shows quantitative analysis of the percentage of cell invasion in various treatment groups. Vertical bars indicate the percentage of cells invading the basement membrane± SEM in each treatment group. * *p* < 0.05 as compared with vehicle-treated controls.

To test the effect of SPA on cell migration, a wound healing assay was performed (Figure 5a). Treatment of the cells with SPA for 48 h caused a dose-dependent inhibition of cell migration in MDA-MB-231, MCF-7, BT-474 and T-47D human breast cancer cells (Figure 5a). The effect of SPA on cell invasion was examined using a transwell chamber assay. As shown in Figure 5b, SPA significantly decreased the level of cell invasion through the matrigel in a dose-dependent manner. Treatment of the highly invasive MDA-MB-231 breast cancer cells with 5, 10 and 20 μM SPA for 24 h inhibited the number of cells invading the lower chamber by 19.1%, 46.3% and 80.8%, respectively (Figure 5b).

### 2.4. Effects of SPA Treatment on Brk Phosphorylation

**Figure 6:**
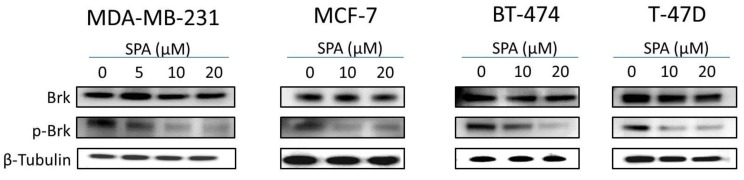
.Western blot analysis showing the SPA treatment effect on total and phosphorylated Brk levels in human breast cancer cells.

To study the effects of SPA treatment on total and phosphorylated Brk levels, Western blot analysis was performed (Figure 6). Results showed a dose-dependent inhibition of Brk phosphorylation after treatment with SPA for 72-h in all tested breast cancer cell lines as compared to their vehicle-treated control group (Figure 6). Meanwhile, SPA treatment had no effects on the total Brk levels in treated cells. 

### 2.5. Effects of SPA Treatment on FAK Phosphorylation

**Figure 7:**
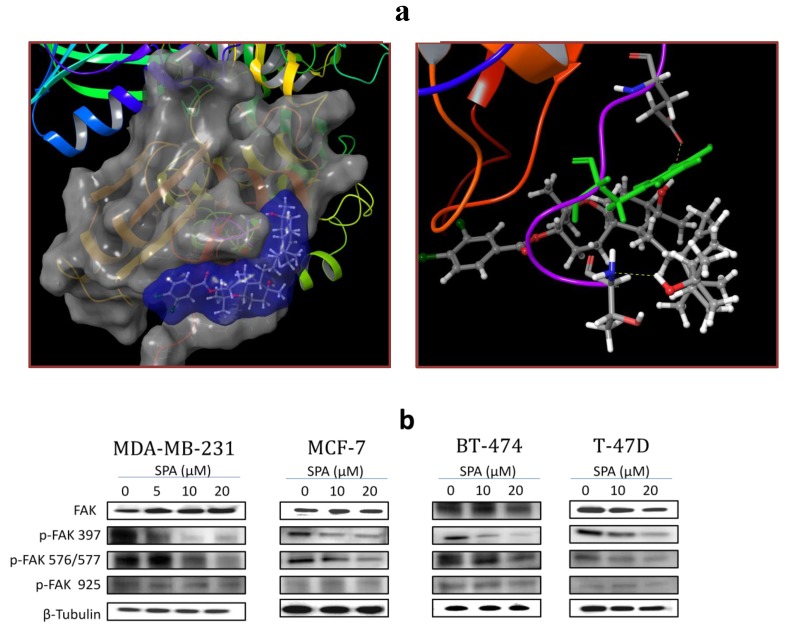
(**a**) (**Left**) Cartoon and solid surface representation of the binding of SPA to Y397 and exceptional fitting at the FAK’s FERM domain binding pocket (PDB code: 2AL6). (**Right**) Interactions of SPA’s C-10 and C-19 OH groups with amino acids E399 and Y394, respectively, at the FAK’s FERM domain binding pocket. SPA’s bicyclodecane ring also forms a hydrophobic interaction with the Y397’s phenyl group, hindering its autophosphorylation, necessary for FAK’s activation. (**b**) Western blot analysis showing the SPA treatment effect on total and phosphorylated FAK levels in human breast cancer cells.

The molecular modeling approach was utilized preliminarily to explore SPA binding at the available crystal structures of FAK. SPA showed no binding affinity at the FAK’s ATP kinase domain. SPA was then docked into the structural pocket of the X-ray crystal structure of the FERM domain of FAK (PDB code: 2AL6), containing the Y397 site using the Glide 5.8 module (2012) of Schrödinger suite in extra-precision (XP) mode. Figure 7a (left panel) shows the best alignment and exceptional fitting of SPA in the FAK’s FERM domain binding pocket. Detailed examination of the SPA binding pose revealed a unique hydrogen bonding interaction between the C-10 OH group with the side chain carbonyl oxygen of E399 (Figure 7a, right panel). In addition, SPA’s C-19 OH group interacts with the backbone amide hydrogen of Y394. Moreover, the bicyclodecane ring forms a hydrophobic interaction with the side chain phenyl of Y397, thus hindering its autophosphorylation necessary for FAK’s activation. The dichlorobenzoate moiety appears to direct the optimal structural orientation necessary for such exceptional fitting at the FAK’s FERM domain binding pocket, which was not matched even with closely related sipholenol A benzoate analogues.

To validate the docking hypothesis, Western blot analysis was performed (Figure 7b). Results showed that SPA treatment for 72-h resulted in a dose-dependent inhibition of FAK phosphorylation at Y397 in all tested breast cancer cell lines, as compared to their vehicle-treated control group. SPA treatment had no effect on the total FAK levels in treated cells. In addition, only higher dose treatment with SPA caused a reduction of FAK phosphorylation at Y576 and Y577; however, none of the SPA treatments resulted in any change in the FAK Y925 phosphorylation.

### 2.6. Effects of SPA Treatment on Brk and FAK Downstream and Upstream Signaling

**Figure 8:**
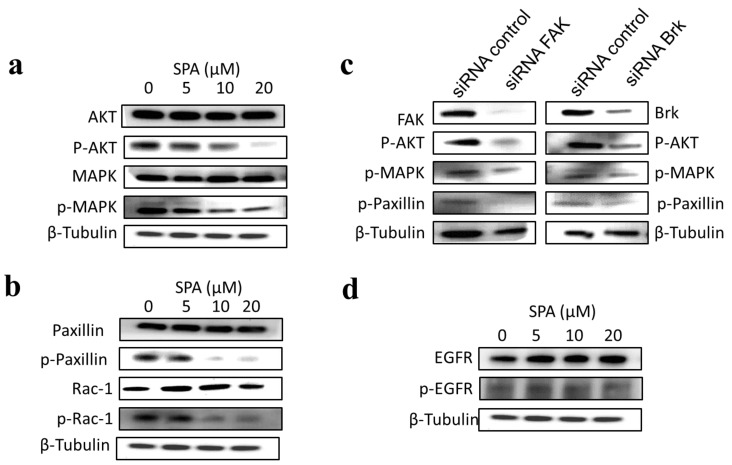
The effects of SPA treatment on the Brk and FAK downstream and upstream signaling molecules in MDA-MB-231 human breast cancer cells. (**a**) Western blot analysis of the total and phosphorylated levels of AKT and MAPK. (**b**) Western blot analysis of the total and phosphorylated levels of paxillin and Rac1. (**c**) Western blot analysis of the cells transfected with a control siRNA, a Brk-targeted siRNA or FAK-targeted siRNA against Brk, FAK, p-AKT, p-MAPK and p-paxillin. (**d**) Western blot analysis of the total and phosphorylated levels of EGFR.

SPA treatment of MDA-MB-231 cells resulted in a dose-dependent suppression of Akt and MAPK phosphorylation (activation) without affecting their total levels (Figure 8a). SPA treatment was associated with a dose-dependent suppression of paxillin and Rac1 phosphorylation, without affecting their total levels (Figure 8b). 

To further confirm the role of Brk and FAK in the activation of the Akt, MAPK and paxillin signaling pathways, siRNAs were used to specifically inhibit Brk and FAK expression in MDA-MB-231 cells. Transfection of Brk-targeted and FAK-targeted siRNAs decreased Brk and FAK protein expressions in this cancer cell line, respectively (Figure 8c). Importantly, Brk and FAK depletion yielded a pattern of pathway inhibition that was remarkably similar to that observed following SPA treatment (Figure 8a–c). Moreover, SPA treatment of MDA-MB-231 cells had little or no effect on the total and phosphorylated EGFR levels (Figure 8d).

**Figure 9:**
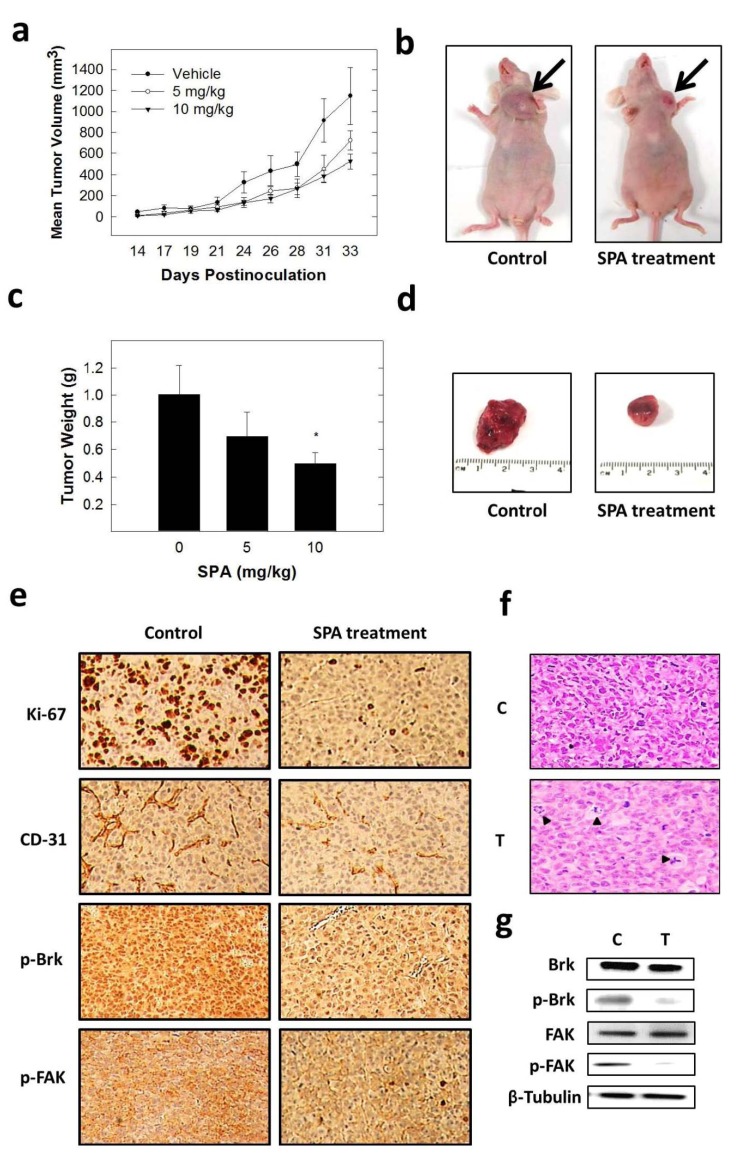
The effect of SPA treatment on tumor growth in the human breast cancer xenograft model. Athymic nude mice with subcutaneous orthotopic MDA-MB-231 human cancer cells were intraperitoneally injected with 5 or 10 mg/kg of SPA or the vehicle control. Treatment regimens were administered 3×/week. (**a**) Tumor volume was evaluated periodically during treatment at the indicated days postinoculation. Points, the mean of tumor volume in mm^3^ of several tumors (*n* = 5) during the course of the treatment period; bars, SEM. (**b**) Two mice harboring human breast cancer. The mouse on the right shows the suppression of tumor growth with SPA treatment (10 mg/kg/day) compared to the vehicle-treated control mouse on the left. (**c**) Vertical bars indicate mean tumor weight at the end of the experiment. *p < 0.05 as compared to the vehicle-treated control. (**d**) Photomicrographs of primary breast tumors from mice with vehicle-treated (**left**) and SPA-treated (10 mg/kg/day) (**right**) cancers. (**e**) Immunostaining of sections obtained from vehicle-treated or SPA-treated (10 mg/kg/day) mice against Ki-67 (mitosis marker), CD31 (endothelial marker), p-Brk and p-FAK antibodies. (**f**) Histopathologic features of mice breast tumors with and without SPA treatment (10 mg/kg/day). In the treatment group, abnormal mitotic figures with fragmented chromosomes were present (arrows). (**g**) Protein expression of total and phosphorylated levels of Brk and FAK in vehicle-treated or SPA-treated (10 mg/kg/day) breast tumors detected by Western blot.

### 2.7. In Vivo Antitumor Activity of SPA

Breast tumor growth was compared between animals with and without SPA treatment. Direct measurement of tumor volume was done starting 14 days after orthotopic tumor inoculation. After SPA treatment, results showed a dose-dependent reduction in tumor volume and tumor weight compared to the vehicle-treated control (Figure 9a–d). In this experiment, 10 mg/kg of SPA caused a reduction in tumor growth by 76%, compared to the vehicle-treated control group, while it had no adverse effect on mice body weight or other clinical symptoms, indicating that SPA lacks potential toxicity in athymic nude mice. Immunohistochemical analysis showed that SPA treatment suppressed mitosis and new vessel formation, as evidenced by the suppression of the expression of their markers, Ki-67 and CD-31, respectively, compared to the vehicle-treated control group (Figure 9e). SPA treatment caused attenuation of p-Brk and p-FAK staining in comparison to the vehicle-control group (Figure 9e). Histopathology studies (H & E staining) have shown an abundance of mitotic nuclei in the control group compared to the SPA-treated group (Figure 9f). Moreover, more cells with abnormal mitosis and fragmented chromosomes were detected in SPA-treated tumors, compared to the vehicle-treated control (Figure 9f). Western blot analysis of isolated tumor tissues showed relatively lower levels of p-Brk and p-FAK (Y397) when compared to the vehicle-treated control group, without any change in their total levels (Figure 9g).

## 3. Discussion

Breast cancer is the second most common form of cancer worldwide and the leading cause of cancer death among women [1]. Although there have been significant improvements in the prevention and diagnosis of this disease, the mortality remains relatively high because of metastatic disease [2]. Tumor cell metastasis includes the detachment of cancer cells from primary tumor, then degrading the basement membrane for the migration and invasion of tumor cells into the peripheral tissue, and finally intravasation into blood or lymphatic vessels and attachment at the target tissue [3]. Among these essential steps, cell migration and invasion mainly contribute to the metastatic spread. Thus, screening novel compounds is an effective way to eradicate high-invasive breast cancer [3].

This study demonstrates hit-to-lead optimization of SPA, a structurally optimized ester derivative of the marine-derived sipholenol A, as a new antitumor entity with a unique ability to interfere with the interplay of Brk and FAK signaling networks, suggesting its therapeutic potential for the control of highly invasive breast cancers. The cross talk between Brk and FAK promotes breast tumor metastasis [7]. In addition to Akt and MAPK signaling, both Brk and FAK control the ability of malignant cells to become invasive by activating paxillin and Rac1 [4,8]. SPA caused a dose-dependent inhibition of the proliferation, migration and invasion of MDA-MB-231, MCF-7, BT-474 and T-47D human breast cancer cells. This suggests the ability of SPA to exert its anticancer effect across a broad spectrum of breast cancer cell lines. In an orthotopic nude mice model, SPA suppressed tumor growth, and this effect was associated with the reduction of Ki-67 (mitosis marker) and CD31 (angiogenesis marker) expression. Findings in this study suggest that SPA is capable of preventing the progression of breast cancer by inhibiting Brk and FAK signaling. 

Breast tumor kinase (Brk) is an intracellular tyrosine kinase and possesses SH3, SH2 and kinase domains, homologues to Src [16]. Brk was identified from a human metastatic breast tumor [16], and subsequent analysis revealed its overexpression in about two-thirds of primary breast tumors, with the highest level in advanced tumors [17]. Brk overexpression positively correlates with the expression of EGFR family receptors [18]. Therefore, Brk stimulates the proliferation of breast tumor cells and mediates epidermal growth factor (EGF)-induced mitogenic and migratory effects [4,5]. Brk associates with the EGF receptor after receptor activation, which subsequently leads to an increased recruitment of PI_3_K and activation of Akt [6]. In addition to stimulating cell growth, Brk is a potent inducer of migration and invasion [4,17]. This function is mediated in part by its phosphorylation of paxillin, which leads to the activation of Rac1 via the adaptor protein, CrkII [4]. Recently, Brk was shown to mediate EGF-induced activation of MAPK, which contributes in part to the proliferation and migration of breast cancer cells in response to these growth factors [19]. 

FAK, a non-receptor protein tyrosine kinase, has been implicated in controlling integrin- and growth factor receptor-mediated biological processes, including cell spreading, motility, migration, differentiation, angiogenesis and survival [20]. FAK regulates cell migration mainly by its auto-phosphorylation at Tyr 397 (Y397), which subsequently phosphorylates other sites of FAK, including its kinase domain, thereby triggering the activation of cell migration signal pathways [20]. Furthermore, FAK functions as a scaffolding protein associating with P130Cas, paxillin and other adaptors to promote cell migration and adhesion [8]. Increased FAK expression and activity are correlated with malignant or metastatic disease and poor patient prognosis [9].

Numerous studies have described the importance of marine natural products to treat human diseases, including cancer [21,22]. Marine natural products are deemed a rich resource of potential chemical molecules exhibiting anticancer properties [10]. Sipholenol A, a marine-derived natural product, was first isolated from the Red Sea sponge *Callyspongia siphonella* in 1983 [11]. Previous studies suggested that the substituted aromatic sipholenol A esters have improved antimigratory activity *versus* unsubstituted aromatic esters [13]. SPA afforded the most active analogue as an anticancer agent. The cell-based *in vitro* activity of SPA was well-correlated with its cell-free Brk phosphorylation inhibitory activity coupled with the absence of any cytotoxicity to the non-tumorigenic MCF10A cells [13]. 

In this study, the inhibitory effect of SPA appears to be mediated through the suppression of Brk and FAK phosphorylation and subsequent inhibition of the phosphorylation of the downstream signaling molecules, such as AKT, MAPK and paxillin. SPA inhibited EGF-dependent mitogenesis, as indicated by a relative large reduction in positive Ki-67 staining in MDA-MB-231 breast cancer cells. Ki-67 is a nuclear antigen localized at the periphery of the chromosome scaffold and nuclear cortex [23]. Ki-67 is expressed in all phases of the cell cycle of proliferating cells (G_1_ phase, S phase, G_2_ phase and M phase), but not cells in the resting phase (G_0_ phase) [23,24,25]. SPA treatment also caused a significant reduction in EGF-induced cell cycle progression, which was accompanied by the reduced expression of cyclin D1 and CDK4 and a corresponding increase in p21 and p27 levels in MDA-MB-231 breast cancer cells. In this study, four breast cancer cell lines representing a wide range of breast tumor phenotypes were used to determine the anticancer effect of SPA. 

SPA treatment caused a dose-dependent inhibition of the cell proliferation and migration of MDA-MB-231, MCF-7, BT-474 and T-47D breast cancer cells, as shown by MTT and wound-healing assays. To study cell invasion, the MDA-MB-231 cell line was used for its aggressive and highly invasive nature [26]. In addition, SPA treatment inhibited Brk phosphorylation in a dose-dependent manner in the four tested breast cancer cell lines with no effect on the total Brk levels. Molecular modeling experiments showed that SPA was docked into the FAK kinase domain without showing any binding affinity. When SPA was docked at the structural pocket of the X-ray crystal structure of the FERM domain of FAK (containing the Y397 site), it showed a perfectly fitting docking pattern. Western blot experiments showed that SPA mainly inhibits the phosphorylation of FAK at the Y397 site, which validates the molecular modeling results. This offered SPA significant advantages because selective blocking of the phosphorylation at the Y397 FAK site consequently will block the activation of several FAK’s downstream signaling. SPA showed little or no effect on the FAK’s ATP-binding site, which shares structural features with several diverse tyrosine kinases, unlike most of other current known small molecule FAK inhibitors. This favors SPA’s uniqueness and selectivity over available FAK inhibitors by avoiding the expected off-target side effects. Furthermore, SPA had little or no activity on other FAK phosphorylation sites. 

The inhibition of Brk and FAK activation caused the inhibition of the activation of downstream signaling molecules, such as AKT, MAPK, paxillin and Rac1. These results were confirmed by siRNA experiments, which showed that the depletion of Brk or FAK in the cells caused a decrease in the levels of p-AKT, p-MAPK, p-paxillin and p-Rac1. Previous studies showed that non-malignant MCF-10A mammary epithelial cells were resistant to SPA antiproliferative effects [13], consistent with the observed tolerance of nude mice to this drug. Intraperitoneal administration (3×/week) of SPA at 10 mg/kg for 33 days in MDA-MB-231 tumor-bearing nude mice did not give rise to overt signs of toxicity. SPA treatment caused a reduction of tumor volume and mitosis, as shown by Ki-67, p-Brk and p-FAK immunostaining and histopathology studies. It also caused a marked reduction in microvessel formation in the tumors. The results in this investigation suggest the value of SPA in the treatment of invasive breast cancer.

## 4. Experimental Section

### 4.1. Chemicals, Reagents and Antibodies

All materials were purchased from Sigma-Aldrich (St. Louis, MO, USA), unless otherwise stated. Sipholenol A-4-*O*-3′,4′-dichlorobenzoate (SPA) was semi-synthesized from sipholenol A according to the method previously described [13]. All antibodies were purchased from Cell Signaling Technology (Beverly, MA, USA), unless otherwise stated. Antibody for Brk was obtained from Abnova (Walnut, CA, USA). Antibodies for p-Brk and Brk siRNA were purchased from Santa Cruz Biotechnology (Santa Cruz, CA, USA). Goat anti-rabbit and goat anti-mouse secondary antibodies were purchased from PerkinElmer Biosciences (Boston, MA, USA). Epidermal growth factor (EGF) was purchased from PeproTech Inc., (Rocky Hill, NJ, USA).

### 4.2. Cell Lines and Culture Conditions

The human breast cancer cell lines, MDA-MB-231, MCF-7, BT-474 and T-47D were purchased from ATCC. The human breast cancer cell line, MDA-MB-231/GFP, was purchased from Cell Biolabs, Inc. (San Diego, CA, USA). The cell lines were maintained in RPMI-1640 supplemented with 10% fetal bovine serum (FBS), 100 U/mL penicillin, 0.1 mg/mL streptomycin in a humidified atmosphere of 5% CO_2_ at 37 °C. SPA was first dissolved in a volume of DMSO to provide a final 25-mM stock solution, which was used to prepare various concentrations of treatment media. The final concentration of DMSO was maintained as the same in all treatment groups within a given experiment and never exceeded 0.1%.

### 4.3. Measurement of Viable Cell Number

The viable cell count was determined using the 3-(4,5-dimethylthiazol-2yl)-2,5-diphenyl tetrazolium bromide (MTT) colorimetric assay. The optical density of each sample was measured at 570 nm on a microplate reader (BioTek, VT, USA). The number of cells/well was calculated against a standard curve prepared by plating various concentrations of cells, as determined using a hemocytometer at the start of each experiment.

### 4.4. Cell Growth and Viability Studies

For growth studies, MDA-MB-231, MCF-7, BT-474 or T-47D cells were plated at a density of 1 × 10^4^ cells per well (6 wells/group) in 96-well culture plates and maintained in RPMI-1640 media supplemented with 10% FBS and allowed to adhere overnight. The next day, cells were washed with phosphate buffer saline (PBS), divided into different treatment groups and then given various treatments in serum-free medium containing 20 ng/mL EGF as a mitogen. Cells in all groups were fed fresh treatment media every other day for a 72-h treatment period. The viable cell number was determined every day using the MTT assay.

### 4.5. Immunocytochemical Fluorescent Staining

MDA-MB-231 cells were seeded on 8-chamber culture slides (Becton Dickinson and Company, Franklin Lakes, NJ, USA) at a density of 3 × 10^5^ cells/chamber (3 replicates/group) and allowed to attach in complete growth media supplemented with 10% FBS overnight. Cells were then washed with PBS and incubated with the vehicle control or treatment media containing 20 ng/mL EGF for 3 days in culture. At the end of the treatments, cells were washed with pre-cooled PBS, fixed with 4% formaldehyde/PBS for 6 min and permeabilized with 0.2% triton X-100 in PBS for 2 min [27]. Fixed cells were washed with PBS and blocked with 5% goat serum in PBS for 1 h at room temperature. Cells were then incubated with Ki-67 mAb, Alexa Fluor^®^ 488 Conjugate (Beverly, MA, USA), overnight at 4 °C in 5% goat serum in PBS. After the final washing, cells were embedded in Vectashield mounting medium with DAPI (Vector Laboratories IN., Burlingame, CA, USA). Fluorescent images were obtained by using a Nikon ECLIPSE TE200-U microscope (Nikon Instruments Inc., Melville, NY, USA). Digital images were captured using Nikon NIS Elements software (Nikon Instruments Inc., Melville, NY, USA). The percentage of cells displaying Ki-67 labeling was determined by counting the number of positive Ki-67-stained cells (green) as a proportion of the total number of cells counted (stained with DAPI, blue). Cells were counted manually in 5 photomicrographs taken randomly in each chamber for every treatment group.

### 4.6. Western Blot Analysis

To study the treatment effects of SPA on MDA-MB-231 tumor cell cycle progression, cells in the various treatment groups were synchronized in the G1 phase [28]. Briefly, MDA-MB-231 cells were plated at a density of 1 × 10^6^ cells/100-mm culture plate in RPMI-1640 media supplemented with 10% FBS and allowed to adhere overnight. Cells were then washed twice with PBS and starved in control or treatment medium containing 0.5% FBS for 48 h to synchronize the cells in the G1 phase. Afterwards, cells were fed various doses of SPA in serum-free-defined media containing 20 ng/mL EGF as the mitogen for 24 h. To study the effect of SPA treatment on EGFR phosphorylation, MDA-MB-231 cells were plated at a density of 1 × 10^6^ cells/100-mm culture plate in RPMI-1640 media supplemented with 10% FBS and allowed to adhere overnight. Cells were then washed twice with PBS and starved in control or treatment medium containing 0.5% FBS for 72 h and stimulated with 100 ng/mL human recombinant EGF for 10 min before cell lysis. In all other Western blot experiments, cells were plated at a density of 1 × 10^6^ cells/100-mm culture plates, allowed to attach overnight, then washed with PBS and incubated in the respective control or treatment in serum-free-defined media containing 20 ng/mL EGF as the mitogen for 72 h. In the case of the *in vivo* experiment, breast tumor tissues were stored at −80 °C until protein extraction. At the end of the treatment period, cells were lysed in RIPA buffer (Qiagen Sciences Inc., Valencia, CA, USA), and breast tumor tissues were homogenized in RIPA buffer using an electric homogenizer (OMNI GLH International, Kennesaw, GA, USA). Protein concentration was determined by the BCA assay (Bio-Rad Laboratories, Hercules, CA, USA). Equivalent amounts of protein were electrophoresed on SDS-polyacrylamide gels. The gels were then electroblotted onto PVDF membranes. These PVDF membranes were then blocked with 2% BSA in 10 mM Tris-HCl containing 50 mM NaCl and 0.1% Tween 20, pH 7.4 (TBST) and then incubated with specific primary antibodies overnight at 4 °C. At the end of the incubation period, membranes were washed 5 times with TBST and then incubated with respective horseradish peroxide-conjugated secondary antibody in 2% BSA in TBST for 1 h at room temperature followed by rinsing with TBST 5 times. Blots were then visualized by chemiluminescence according to the manufacturer’s instructions (Pierce, Rockford, IL, USA). Images of protein bands from all treatment groups within a given experiment were acquired using a Kodak Gel Logic 1500 Imaging System (Carestream Health Inc., New Haven, CT, USA). The visualization of β-tubulin was used to ensure equal sample loading in each lane. All experiments were repeated at least 3 times.

### 4.7. Analysis of Cell Cycle Progression by Flow Cytometry

To study treatment effects on the cell cycle, MDA-MB-231 cells were plated, then synchronized in the G1 phase and fed treatments as described above. At the end of the experiment, cells in the various treatment groups were isolated with trypsin and then resuspended in ice-cold PBS, fixed with cold (−20 °C) 70% ethanol and stored at 4 °C for 2 h. Afterwards, cells were rehydrated with ice-cold PBS and then incubated with DNA staining buffer (sodium citrate 1 mg/mL, triton-X 100 3 μL/mL, propidium iodide 100 μg/mL, ribonuclease A 20 μg/mL) for 30 min at 4 °C in the dark. DNA content was then analyzed using a FACSCalibur flow cytometer (BD Biosciences, San Jose, CA, USA). For each sample, 10,000 events were recorded, and histograms were generated using CellQuest software (BD Biosciences, San Jose, CA, USA). All experiments were repeated at least three times.

### 4.8. Wound-Healing Assay

The *in vitro* wound-healing assay was used to assess directional cell motility in two dimensions. The cell migration assay was carried out in 24-well plates using Ibidi^®^ 2-chamber inserts (Martinsried, Germany). Chambers were added to the wells and then seeded with typically 30,000–70,000 cells to achieve a confluent monolayer overnight. Treatments were also added at this time. The inserts were removed after overnight culture to reveal patches of cells separated by a 500-mm gap. Fresh medium containing treatments was added and migration was allowed to proceed. Wound healing was then visualized at 0 and 48 h by a Nikon ECLIPSE TE200-U microscope (Nikon Instruments Inc., Melville, NY, USA). Digital images were captured using Nikon NIS Elements software (Nikon Instruments Inc., Melville, NY, USA) (Figure 5a). The distance traveled by the cells was determined by measuring the wound width at 48 h and subtracting it from the wound width at the start of treatment (*t*_0_, time zero). The values obtained were then expressed as the percent of migration, setting the gap width at *t*_0_ as 100%. Each experiment was done in triplicate, and the distance migrated was calculated in three or more randomly selected fields per treatment group.

### 4.9. Cell Invasion Assay

MDA-MB-231 cell invasion was determined using the CytoSelect™ Cell Invasion Assay (Cell Biolabs, Inc., San Diego, CA, USA) according to the manufacturer’s instructions (Figure 5b). Briefly, MDA-MB-231 cells were pre-treated with SPA for 24 h. Basement membranes of Boyden chambers were rehydrated with 300 μL serum free RPMI-1640, and 3 × 10^5^ cells were then seeded into the upper area of the chamber in serum-free RPMI-1640. Bottom wells were filled with defined control serum-free media supplemented with 20 ng/mL EGF containing SPA or no SPA. After 24 h of incubation (37 °C, 5% CO_2_), non-invasive cells were removed from the upper chamber, and cell invasion was assessed by light microscopy after the staining of invaded cells with crystal violet Cell Stain Solution (Cell Biolabs, San Diego, CA, USA). For the colorimetric quantification of invasion, inserts were then placed in extraction buffer (200 μL, 10 min), and absorbance at 560 nm was determined after transfer to a 96 well plate (100 μL per well) using a BioTek microtiter plate reader (BioTek, Winooski, VT, USA).

### 4.10. RNA Interference

Transfection of small interfering RNA (siRNA) into cells was conducted when the cells reached 70% confluence. Experiments were conducted using Lipofectamine^®^ RNAiMAX Reagent (Life Technologies, Grand Island, NY, USA) as a transfection agent and siRNAs for Brk, FAK and the control. Experiments were conducted according to the manufacturers’ instructions.

### 4.11. Molecular Modeling

The *in silico* experiments were carried out using the Schrödinger molecular modeling software package installed in a workstation running on an Intel^®^ CORE™ 2 Duo processor (Dell, TX, USA) with 16 GB RAM and Windows 7 Enterprise as the operating system.

#### 4.11.1. Protein Structure Preparation

The X-ray crystal structure of the FERM domain of the focal adhesion kinase (PDB code: 2AL6) [29] was retrieved from the Protein Data Bank [30]. The Protein Preparation Wizard of the Schrödinger suite was implemented to prepare the FERM domain of FAK [31]. The protein was reprocessed by assigning bond orders, adding hydrogens, creating disulfide bonds and optimizing H-bonding networks using PROPKA (Jensen Research Group, Copenhagen, Denmark). Missed side chains for amino acid residues of Chain A (Q363, Y394) and Chain B (K375, Y394) were detected and added using Prime version 3.1. Finally, energy minimization with a root mean square deviation (RMSD) value of 0.30 Å was applied using an Optimized Potentials for Liquid Simulation (OPLS_2005, Schrödinger, New York, NY, USA) force field.

#### 4.11.2. Ligand Structure Preparation

The structure of SPA was sketched in the Maestro 9.3 panel (Maestro, version 9.3, 2012, Schrödinger, New York, NY, USA). The Lig Prep 2.3 module (Lig Prep, version 2.3, 2012, Schrödinger, New York, NY, USA) of the Schrödinger suite was utilized to generate the 3D structure and to search for different conformers. The Optimized Potentials for Liquid Simulation (OPLS_2005, Schrödinger, New York, NY, USA) force field was applied to geometrically optimize the ligand and to compute partial atomic charges. Finally, at most, 32 poses per ligand were generated with different steric features for the subsequent docking study.

#### 4.11.3. Molecular Docking

The prepared FERM domain of FAK was employed to generate receptor energy grids using the default value of the protein atomic scale (1.0 Å) within the cubic box centered on the Y397 site. After receptor grid generation, the prepared ligand was docked using the Glide 5.8 module (Glide, version 5.8, 2012, Schrödinger, New York, NY, USA) in extra-precision (XP) mode [32].

### 4.12. Xenograft Animal Model

All animal experiments were approved by the Institutional Animal Care and Use Committee, University of Louisiana at Monroe, and were handled in strict accordance with good animal practice as defined by the NIH guidelines. Athymic nude mice (Foxn1^nu^/Foxn1^+^, 4–5 weeks, female) were purchased from Harlan (Indianapolis, IN, USA). The mice had free access to standard pellet food and water. The animals were acclimated to animal house facility conditions at a temperature of 18–25 °C, with a relative humidity of 55% to 65% and a 12-h light/dark cycle, for one week prior to the experiments. MDA-MB-231/GFP human breast cancer cells were cultured and resuspended in serum-free DMEM medium (20 μL). For anesthesia, ketamine + xylazine (1.0 mL xylazine (20 mg/mL) was added to 10.0 mL ketamine (100 mg/mL) to make 11.0 mL at 92 mg/mL of stock) was used. Then, 1.0 mL of this solution was diluted with 9.0 mL sterile normal saline to make a 9.2 mg/mL solution. Of this solution, 10 mL/kg was used, which is equivalent to 10 μL/g. After anesthesia, cell suspensions (1 × 10^6^ cells/20 μL) were inoculated into the second mammary gland fat pad just beneath the nipple of each animal to generate orthotopic breast tumors. At 48 h post-inoculation, the mice were randomly divided into three groups: (i) vehicle-treated control group (*n* = 5), (ii) 5 mg/kg SPA-treated group (*n* = 5), (iii) 10 mg/kg SPA-treated group (*n* = 5). Treatment (3×/week) started 5 days postinoculation with intraperitoneal (i.p.) administered vehicle control (DMSO/saline) or treatment. Selection of this dose was based on preliminary *in vivo* data on SPA. The mice were monitored by measuring tumor volume, body weight and clinical observation. Tumor volume (V) was calculated by V = L/2 × W^2^, where L was the length and W was the width of tumors. All the mice were sacrificed at day 33 postinoculation, and the tumors were excised and weighed. Breast tumor tissues were stored at −80 °C until total protein extraction for Western blot analysis or stored in 70% ethanol at RT for immunohistochemistry and histopathology studies.

### 4.13. Immunohistochemistry and Histopathology

Breast tumor tissues were taken for light microscopy with H&E staining. The H&E was a standard procedure that was run on an automated Sakura Prisma unit. The tumor specimens were processed with the use of alcohols and xylene and then infiltrated in paraffin wax using the Excelsior™ ES Tissue Processor. Paraffin sections were dewaxed in xylene, rinsed in grade alcohol, rehydrated in water, then placed in citric buffer (PH 6.0) and treated in a microwave oven with high power for 3 min and 10% goat serum for 30 min. Subsequently, antibodies with a proper dilution were applied on the sections as follows: CD31 (Pierce Product# PA5-32321; 1:50 dilution, 1 h at RT), Ki-67 (Cell Signaling Product# #9027; 1:150 dilution, 1 h at RT), p-Brk (Bioss Product# bs-12890R; 1:100, 1 h at RT) and p-FAK (Cell Signaling Product# #8556; 1:100, 1 h at RT). Following that, secondary antibodies (Ventana Multimer Anti Rb-HRP Product#760-4311 24 min at RT) were applied. Signals were developed with Vector ImmPACT DAB Product#SK-4105 for 8 min at RT. The sections were finally counter stained by hematoxylin solution for 1 min at RT. 

### 4.14. Statistics

The results are presented as the means ± SEM of at least three independent experiments. Differences among various treatment groups were determined by the analysis of variance (ANOVA) followed by Dunnett’s test using PASW statistics^®^ version 18 (Quarry Bay, Hong Kong). A difference of *p* < 0.05 was considered statistically significant as compared to the vehicle-treated control group. The IC_50_ values (concentrations that induce 50% cell growth inhibition) were determined using a non-linear regression curve fitting analysis using GraphPad Prism software version 6 (La Jolla, CA, USA).

## 5. Conclusions

In conclusion, SPA has shown its great potential in the suppression of breast tumor growth and invasion through the inhibition of Brk and FAK signaling. In addition, it causes cell cycle arrest in the G_1_ phase and decreases microvessel formation in the tumors. These results suggest that SPA may be a promising candidate for potential therapies for treating invasive breast cancer.

## Abbreviations

ER: estrogen receptor
EGFR: epidermal growth factor receptor
HER-2: human epidermal growth factor receptor 2
DAPI: 4′,6-diamidino-2-phenylindole
PI: propidium iodide
PDB: protein data bank
H & E: hematoxylin and eosin
ATCC: American Type Culture Collection
GFP: green fluorescence protein
RPMI: Roswell Park Memorial Institute
DMSO: dimethyl sulfoxide
FBS: fetal bovine serum
RIPA: radioimmunoprecipitation assay
BCA: bicinchoninic acid assay
PVDF: polyvinylidene fluoride
BSA: bovine serum albumin
NIH: National Institutes of Health
HRP: horseradish peroxidase
DAB: 3,3′-diaminobenzidine
